# Low Grade Endometrial Stromal Sarcoma: A Case Report

**Published:** 2015-01

**Authors:** Reena Jain, Swaraj Batra, Ayesha Ahmad, Arifa Anwar Elahi, Monika Gupta, Poonam Saith

**Affiliations:** Department of Obstetrics and Gynaecology, Hamdard Institute of Medical Sciences and Research, Jamia Hamdard, Hamdard Nagar, New Delhi, India

**Keywords:** Endometrial stromal sarcoma, Uterine leiomyoma, Immunohistochemistry

## Abstract

Endometrial stromal sarcoma (ESS) is a rare malignant tumor of the endometrium, occurring in the age group of 40–50 years. We report a case of low-grade ESS in a 39-year-old woman, presenting as rapid enlargement of a uterine fibroid polyp associated with irregular and excessive vaginal bleeding. Polypectomy followed by pan hysterectomy was performed. Histopathological examination and immunohistochemistry confirmed LGESS. As the tumor is rarely encountered, management protocols are still questionable. In our case, we tried a different post-surgical protocol and the patient is being closely followed up. Although rare, ESS should be considered in the differential diagnosis of all women who present with a rapid enlargement of a uterine leiomyoma.

## Introduction

Sarcoma is a rarely encountered malignancy of uterus, with an incidence of 1-2 cases per 100,000 women. The site of origin may be connective tissue, smooth muscle, or endometrial stromal. The latter (endometrial stromal sarcoma, ESS) is still rarer tumors that make up approximately 10% of all uterine sarcomas.^1^

The World Health Organization (WHO) classifies endometrial stromal tumors as benign endometrial stromal nodule (ESN) and ESS. ESNs are termed benign, as they do not infiltrate myometrium. In contrast, ESSs are characterized by infiltration of myometrium. The histopathology reveals uniform small cells bearing resemblance to the proliferative stage endometrial stroma. ESSs are classified based on cell morphology and mitotic count into either low-grade (LGESS) or high-grade (HGESS) tumors.^2^

In comparison with HGESS, the age group of LGESS is usually younger (45-55 years). LGESS demonstrates less frequent mitosis (<3 per 10 high-power fields) and there is no associated hemorrhage or necrosis. The pathogenesis of these tumors is yet to be delineated. Identified risk factors are past exposure to pelvic radiation therapy, long-term tamoxifen use, and unopposed estrogen use. We report a case of low-grade ESS presenting as rapid enlargement of a uterine leiomyoma. 

## Case Report

A 39-year-old woman P_2+0 _of lower middle class background presented with polymenorrhagia for eight months, and retention of urine for two days. USG done six months earlier showed a small intramural leiomyoma 2.8×2.2 cm in lower uterine body. Dilatation and curettage (D&C) had done 6 months earlier, revealed secretory endometrium on histopathological examination (HPE).

**Figure 1:**
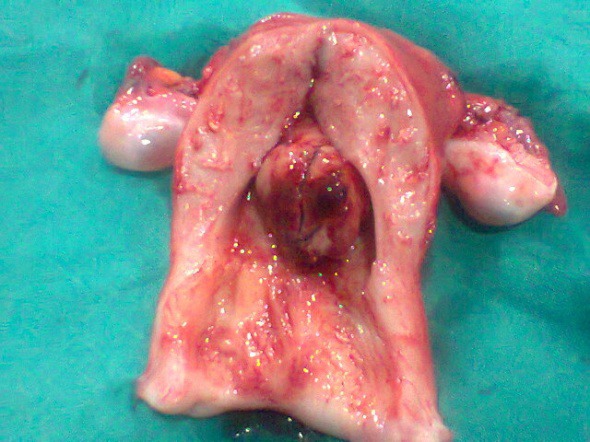
Cut Section of the uterus showing a polypoidal growth arising from endometrium, 5.0×4.5 cm with areas of hemorrhage. Myometrium is thickened and multiple calcifications are present. Ovaries are multicystic.

**Figure 2:**
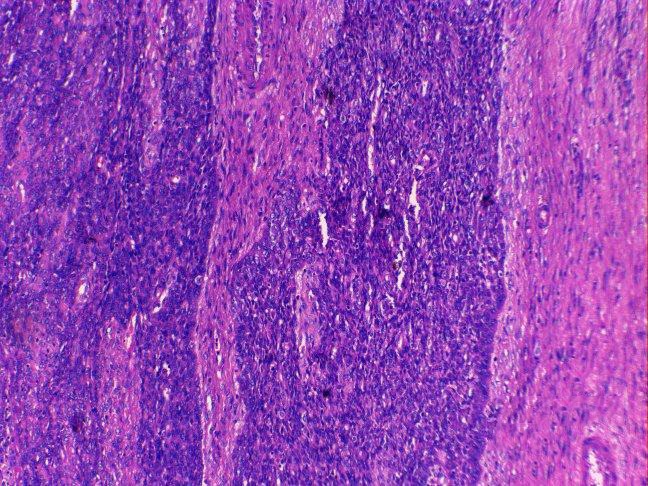
Endometrial stromal cells positive for CD-10 stain (internal control).

**Figure 3:**
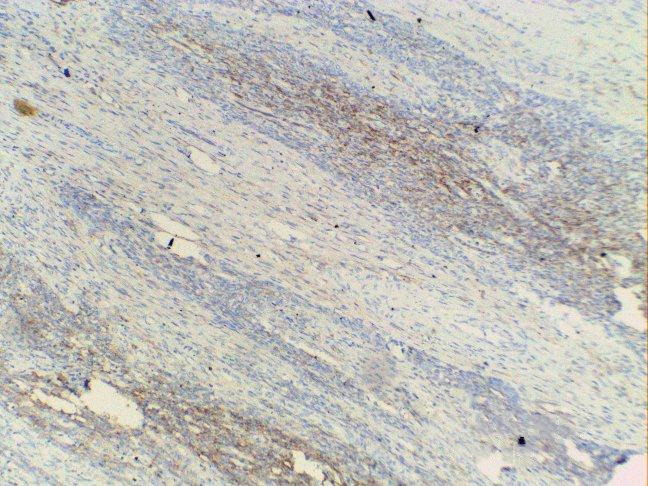
CD 10 positvity focally in tumor cels.

Her last childbirth was 11 years back and she was ligated. The patient was pale and hemoglobin (Hb) was 6 gm%. Vitals were stable and systemic examination was unremarkable. On speculum examination, a large retort shaped shaggy mass about 3 inches in diameter was seen coming out through os. On vaginal examination, the mass was felt as firm, globular and filling vagina in its upper part. Cervical rim felt all around the pedicle was high up. Uterus was 8 weeks in size, soft, anteverted. Bilateral fornices were clear. Pelvic ultrasound (USG) showed a cervical mass lesion with well-defined outline 9×8×8 cm, with heterogeneous echo pattern and multiple hyperechoic lesions in it. Endometrial cavity was pushed anteriorly. Uterus was bulky, bilateral adnexa were normal. Mild hydronephrotic changes were present in the left kidney. An impression of cervical leiomyoma was made. One unit of blood was transfused preoperatively and cervical polypectomy with endometrial curettage was done. The polyp was friable, globular and had a ragged appearance. It measured around 6.0×5.5×4.0 cm. Cut section showed a fleshy greyish pink appearance. On HPE, a differential diagnosis of ESN and LGESS was made and the patient was posted for laparotomy. Uterus was enlarged to 8 weeks size and soft in consistency. Bilateral ovaries were cystic as shown in figure 1). Total abdominal hysterectomy with bilateral salpingo-oophorectomy was done. Pelvic lymph nodes were not enlarged. There were no metastases in the abdomen or extension in the broad ligament. Cut section revealed a nodular vascular mass 3.5×4.0 cm inside the endometrial cavity. Diffuse nodularity was present in entire myometrium. HPE and immunohistochemistry with CD10 (figure 2 and 3) confirmed the diagnosis of LGESS. According to the new 2009 FIGO Staging, it was stage IB disease. Postoperative period was uneventful and the patient was discharged after 5 days.

The patient has been put on injection leuprolide acetate (3.75 mg) subcutaneously every 28 days for six months. At follow visit after 3 months, we performed a Pap smear, chest X-ray, and pelvic ultrasound. All investigations were normal. We plan to repeat clinical examination and the same investigations after 6 months and thereafter the patient will be followed up annually. As there are no established guidelines for the management of ESS because of the rarity of cases, we suggest that all cases with a diagnosis of ESS should be reported so that a proper protocol for management can be drawn up. 

## Discussion

ESS is an occasional diagnosis in a patient presenting as leiomyoma uterus. The symptoms are nonspecific, mostly abnormal uterine bleeding. An early diagnosis is essential because patient survival is directly related to tumor stage. Uterine sarcomas most often affect postmenopausal women. Women with LGESS are younger with a median age of 45 and 55 years. Our patient presented at 39 years, which is a rarity in itself. The presenting features are similar to uterine leiomyoma; abnormal vaginal bleeding pelvic mass or abdominal pain and pressure caused by an enlarging pelvic mass, and some patients may be asymptomatic.^3^ The index case presented with abnormal uterine bleeding and urinary retention. While often indolent in behavior, ESS is malignant and can spread to the vagina, fallopian tubes, ovaries, bladder and ureters. Distant metastasis to lung, heart and to other sites has also been reported.^4^^,^^5^ Up to 30% of women with low grade ESS have an extra uterine disease at presentation. Preoperative diagnosis is often difficult and around 75% are diagnosed as benign leiomyoma. Endometrial curettage and HPE do not help due to similarity with normal endometrium.^6^ Besides, the tumor has a propensity to grow through intramural sections of the uterus instead of intracavitary part. This prevents accurate histopathological diagnosis preoperatively. Ultrasound and magnetic resonance imaging are inconclusive and the diagnosis is usually uterine leiomyoma or pelvic mass.

A case of ESS was reported in a 30-year-old woman with ultrasound and Doppler findings suggestive of leiomyoma uterus. Sarcomatous change was suspected in view of rapid enlargement of the tumor. Endometrial aspiration revealed secretory endometrium with neoplastic cells and hysterectomy was done.^7^ Another rare presentation of LGESS was as a low grade endometrial sarcoma of endocervix presenting as a soft hemorrhagic mass in the posterior cervix looking like a degenerated leiomyoma.^8^ At times it is very difficult to differentiate ESS from cellular leiomyoma. In these cases, immunohistochemistry is especially helpful to arrive at the final diagnosis. The immunohistochemical markers such as h-caldesmon and CD 10 may solve the diagnostic problem as CD 10 staining is positive in ESS but not in leiomyoma. We performed CD 10 staining to establish the diagnosis.

The definitive management of ESS is surgical, both to establish the diagnosis as well as for treatment. At surgery, an enlarged uterus with soft yellow necrotic and hemorrhagic tumor (especially with worm like extensions into pelvic veins) serves as a pointer for diagnosis. The presence of large, thick walled muscular vessels can be used to distinguish a highly cellular leiomyoma from stromal proliferation.^9^ The definitive treatment is total abdominal hysterectomy, bilateral salpingo-oophorectomy and excision of all grossly detectable tumor.^10^ Local recurrences occur in 70% of cases. Postoperative radiotherapy or progesterone is an effective adjuvant treatment providing high local control rate in uterine sarcomas. Hormone therapy with medroxyprogesterone, tamoxifen, gonadotropin releasing hormone (GnRH) analogues and aromatase inhibitors are suggested for LGESS stage 3-4 and for recurrent disease.^11^^,^^12^ In a study by Chu and colleague, 75% of patients with stage 1 disease did not recur if treated with adjuvant medroxyprogesterone acetate compared with 29% similar stage patients who did not receive.^13^ We have administered GnRH to our patient and at 3 months follow up she was doing well.

## Conclusion

ESS is a rare malignant tumor, presenting as abnormal uterine bleeding in perimenopausal women. The usual preoperative diagnosis is uterine leiomyoma and definitive diagnosis is achieved only after histopathology of uterus. By reporting our case, we wish to stress the necessity for a high degree of suspicion to diagnose this tumor even in younger women. A prompt diagnosis and timely intervention are keys to improve patient survival. 

## References

[1] Ashraf-Ganjoei T, Behtash N, Shariat M, Mosavi A (2006). Low Grade Endometrial Stromal Sarcoma of uterine corpus, a clinic-pathological and survey study in 14 cases. World J Surg Oncol.

[2] Leung F, Terzibachian JJ, Aouar Z, Govyadovskiy A, Lassabe C (2008). Uterine Sarcomas: clinical and histopathological aspects. Report on 15 cases. Gynecol Obstet Fertil.

[3] Fekete PS, Vellios F (1984). The clinical and histologic spectrum of endometrial stromal neoplasm: a report of 41 cases. Int J Gynecol Pathol.

[4] Matsuura Y, Yasungag K, Kuroki H, Inagaki H, Kashimura M (2004). Low-grade endometrial stromal sarcoma recurring with multiple bone and lung metastases: report of a case. Gynecol Oncol.

[5] Aubry MC, Myers JL, Colby TV, Leslie KO, Tazelaar HD (2002). Endometrial Stromal Sarcoma Metastatic to the lung: A detailed analysis of 16 patients. Am J Surg Pathol.

[6] Jin Y, Pan L, Wang X, Dai Z, Huang H, Guo L (2010). Clinical characteristics of endometrial stromal sarcomafrom an academic medical hospital in china. Int. J Gynecol Cancer.

[7] Puliyath G, Nair VR, Singh S (2010). Endometrial stromal sarcoma. Indian J Med Paediatr Oncol.

[8] Hasiakos D, Papakonstantinou K, Kondi-Paphiti A, Fotiou S (2007). Low-grade Endometrial stromal sarcoma of the endocervix. Report of a case and review of the literature. Eur J Gynaecol Oncol.

[9] Olive E, Clement PB, Young RH (2000). Endometrial stromal tumours. An update on a group of tumors with a protean phenotype. Adv Anat Pathol.

[10] Gadducci A, Cosio S, Romanini A, Genazzani AR (2008). The management of patients with uterine sarcoma: a debated clinical challenge. Crit Rev Oncol Hematol.

[11] Palombaa S, Falboa A, Mocciaroa R, Russob T, Zulloa F (2009). Laparoscopic treatment for endometrial cancer: A meta-analysis of randomized controlled trials (RCTs). Gynecol Oncol.

[12] Lindner T, Pink D, Kretzschmar A, Mrozek A, Thuss-Patience PC, Reichardt P (2005). Hormonal treatment of endometrial stromal sarcoma: a possible indication for aromatase inhibitors. J Clin Oncol.

[13] Chu MC, Mor G, Lim C, Zheng W, Parkash V, Schwartz PE (2003). Low grade endometrial stromal sarcoma: hormonal aspects. Gynaecol Oncol.

